# Valeurs spirométries de référence dans la population bantoue de Kinshasa de 20 à 70 ans

**DOI:** 10.11604/pamj.2019.33.295.16843

**Published:** 2019-08-13

**Authors:** Boniface Muamba Kamanga, Jean Marie Ntumba Kayembe, Constant Ekisawa Nkiama, Patrick Kalambayi Kayembe, Louise Kalabo Kikontwe, Marie Jeanne Lenga Nkoy

**Affiliations:** 1 Unité Cardio-respiratoire, Département de Médecine Physique, Faculté de Médecine, Université de Kinshasa, Kinshasa, République Démocratique du Congo; 2 Service de Pneumologie, Département de Médecine Interne, Faculté de Médecine, Université de Kinshasa, Kinshasa, République Démocratique du Congo; 3 Ecole de santé publique, Faculté de Médecine, Université de Kinshasa, Kinshasa, République Démocratique du Congo

**Keywords:** Valeurs pyrométriques, référence, Kinshasa, Spirometry values, reference, Kinshasa

## Abstract

**Introduction:**

Les valeurs spirométriques de référence ne sont pas extrapolables entre populations, étant tributaires de nombreuses variables humaines et environnementales, d'où l'intérêt des études locales dans ce domaine. L'objectif est de déterminer des valeurs de référence chez des adultes sains de Kinshasa.

**Méthodes:**

Une étude transversale incluant 7443 sujets (3208 femmes, 43%). Le VEMS, la CVF et le DEP ont été corrélés aux données anthropométriques. Cinq groupes d'âge ont été constitués et les comparaisons effectuées en fonction du sexe, de l'âge, de l'IMC et de la pratique d'une activité sportive.

**Résultats:**

Les différences sont évidentes entre sexes, pour le VEMS (3,00 vs 2,21 L), la CVF (3,19 vs 2,38 L), et le DEP (6,8 vs 5,70 L/s); de même que pour les tranches d'âge extrêmes. Elles sont comprises entre: 2,33 et 4,54 vs 1,93-3,3 L dans le groupe de 20-29 ans et 1,76-3,39 vs 1,60 vs 2,53 L chez les 60-70 ans; pour La CVF entre 2,44-4,89 vs 1,96-3,56 L et 1,79-3,78 vs 1,66-2,74 L; pour le DEP entre 4,34-12,2 vs 3,62-8,58 L/s et 2,99-6,76 vs 2,99-7,34L/s chez les 60-70 ans.

**Conclusion:**

Les différences liées au genre, à l'âge, aux données anthropométriques ainsi qu'à la pratique d'une activité sportive sont évidentes. Ces résultats justifient des enquêtes plus étendues et montrent la pertinence des valeurs basées sur les percentiles dans la déterminantion d'un référentiel spirométrique dans une population donnée.

## Abstract (en)

**Introduction:**

Spirometric reference values cannot be extrapolated in the populations, being subject to many environmental and human variables; hence the interest of local studies in this field.

**Methods:**

This study aims to determine the reference values among healthy adults in Kinshasa. We conducted a cross-sectional study including 7443 subjects (3208 women, 43%). FEV1, FVC, PEF were correlated with anthropometric data. Five age groups were formed and comparisons were made on the basis of sex, age, BMI and of the practice of sporting activity.

**Results:**

Differences are evident between the sexes, in terms of FEV1 (3.00 vs 2.21 L), FVC (3.19 vs 2.38) and the PEF (6.8 vs 5.70 L/s); same as for the outer age categories. FEV1 ranged between 2.33 et 4.54 vs 1.93-3.3 L in the age group 20-29 years and 1,76-3,39 vs 1,60 vs 2,53 L in the age group 60-70 years; FVC ranged between 2,44-4,89 vs 1,96-3,56 L and 1,79-3,78 vs 1,66-2,74 L ; PEF ranged between 4,34-12.2 vs 3,62-8.58 L/s and 2.99-6.76 vs 2.99-7.34L/s in the age group 60-70 years.

**Conclusion:**

The differences related to gender, age, anthropometric data as well as to the practice of sporting activity are obvious. These results warrant further and more extended investigations and show the relevance of values based on percentiles in the determinantion of a spirometry standard in a given population.

## Introduction

Les tests d'exploration respiratoire sont couramment utilisés dans l'orientation du diagnostic et le suivi des affections du système respiratoire. Leur extension dans la surveillance et l'appréciation du niveau d'aptitude cardiorespiratoire chez les sportifs, ainsi que dans l'évaluation des patients en réhabilitation respiratoire, est de plus en plus recommandée [1]. De nombreux facteurs influencent les valeurs des volumes et débits respiratoires, rendant difficile les efforts de standardisation des normes à l'échelle planétaire. C'est le cas de l'âge, du sexe, de la race et de la morphologie, cette dernière étant sous la dépendance de l'hérédité, de l'activité hormonale, des données climatologiques et du statut économique [2-7]. Il est donc évident d'envisager les limites des normes établies sur une population donnée, dans un espace géographique bien déterminé dans l'interprétation des valeurs spirométriques au niveau mondial [4]. Si la difficulté d'un consensus au niveau des individus sains apparait comme une évidence, on comprend davantage la difficulté de cette interprétation en situation pathologique. L'American Thoracic Society (ATS), la Société de Pneumologie de Langue Française (SPLF) et la Société Belge de Pneumologie ont entrepris, à travers des panels d'experts, des travaux en vue d'adopter des techniques et des valeurs les mieux standardisées possibles [1-3]. Par exemple, le critère spirométrique d'appréciation de l'obstruction bronchique recourt aujourd'hui au rapport VEMS / CVF en dessous du 5^ème^ percentile, plutôt qu'à une valeur de ce rapport en dessous de 70% comme recommandé antérieurement [6]. Les normes de référence des valeurs spirométriques d'un continent ne pourraient donc être extrapolées à une autre population, sans risque de biais d'estimations. Des données des pays développés d'Amérique latine [7, 8], d'Amérique du Nord [9-15]; d'Asie [16-19] et d'Europe ([1-6], [20-24]) sont largement disponibles. Celles disponibles sur le continent africain proviennent essentiellement des équipes d'Algérie [25], d'Afrique du Sud [26], de Tunisie [27], du Sénégal [28], du Soudan [29], de Madagascar [30], du Ghana [31], du Rwanda [32], et du Maroc [33]. Dedoyard *et al.* en 1972 [34], Ghesquiere *et al.* en 2009 [35], sont les premiers à rapporter dans une population estudiantine de Kinshasa, notamment des différences interraciales. L'équipe de Kamanga *et al.* [36], dans la même ville, a entrepris en 2013, une étude incluant 1004 sujets réputés sains et d'âge compris entre 18 et 70 ans, ayant abouti à proposer des valeurs spirométriques locales, intégrant des données anthropométriques et écologiques, selon les recommandations des sociétés savantes [37]. La modicité des données locales, qui du reste n'avaient pas intégré l'impact de la pratique du sport sur les paramètres individuels, justifie la présente enquête dont l'objectif principal était de déterminer les valeurs spirométriques de référence susceptibles d'utilisation dans la pratique médicale ou en médecine sportive et professionnelle.

## Méthodes

**Nature et cadre de l'étude:** l'enquête transversale analytique a été réalisée de mars 2014 à Octobre 2016 dans la ville de Kinshasa. Seize communes sur 24 ont été choisies en fonction de leur densité de population et leur situation géographique, selon une méthode d'échantillonnage en grappes (commune, quartier). Au total, 7443 sujets 3208 (43%) femmes et 4235 (57%) hommes; sex-ratio= 1,3 ont été inclus dans l'étude. Ils appartenaient tous à l'ethnie bantoue, et étaient reconnus non-fumeur ou ex-fumeur (sevré depuis au moins 6 mois. Ils devaient être indemnes de symptômes ou maladies respiratoires, de symptômes ou maladies du système cardiovasculaire, de déformation visible de la cage thoracique ou de la colonne vertébrale. Seuls les sujets âgés entre 20 et 70 ans, habitant la ville de Kinshasa depuis au moins 6 mois étaient éligibles. Les sujets avaient été tirés des 16 communes, quartiers et avenues suivant la technique d'échantillonnage de Lemeshow reprise dans le livre de Harly J [37]. Ils ont ensuite été regroupés en 5 classes d'âge comme illustré dans le Tableau 1.

**Tableau 1:** Répartition des sujets selon les catégories d’IMC des hommes et femmes

IMC	Femmes (n=3208)	Hommes (n=4235)
Maigre (< 18)	224(7,0)	301(7,1)
Normale (18.0-24.9)	1388(43,3)	2941(69,0)
Surpoids (25.0-29.9)	1595(49,7)	1021(24,0)

Maigre: <18 Kgm^2^; normale: 18.0-24.9 Kgm^2^; surpoids: 25.0- 29.9 Kgm^2^.

**Paramètres anthropométriques:** les mesures du poids, à l'aide d'une balance de marque OMRON et celle de la taille à l'aide d'une toise de marque SECA, ont été effectuées et consignées pour chaque enquêté. L'impédancimétrie, utilisant une balance munie d'un moniteur de la composition corporelle de marque OMRON, type BF 511 sur batteries renouvelables, (fabriquant OMRON HEALTHCARE Co Ltd, Kyoto, Japon) avait permis de déterminer le poids et automatiquement, de fournir l'IMC. Le périmètre thoracique (PT), le tour de taille (TT) et le tour de hanche (TH) avaient été également mesurés selon les conventions usuelles en la matière (respect des repères) avec un ruban métrique: le périmètre thoracique était mesuré à hauteur des mamelons pour les hommes et en dessous des seins pour les femmes; et le tour de taille, à 5 cm au-dessus de l'ombilic. Pour la mesure du tour de hanche, le mètre ruban passait par les grands trochanters et sur la partie la plus proéminente de la fesse. Le rapport tour de taille/tour de hanche (RTH) était également calculé et enregistré.

**Paramètres spirométriques:** les tests spirométriques ont été effectués à l'aide d'un spiromètre de poche SPIROBANK A23-0U sur batteries renouvelables (Constructeur: MIR via Magglolino, Rome-Italie). Les mesures enregistrées concernaient: le VEMS (en litres), la CVF (en litres), et le DEP (en litres/s); ces valeurs ont permis le calcul de l'indice de Tiffeneau (VEMS/CVF*100). Le protocole de mesures a scrupuleusement respecté les recommandations des sociétés savantes [1-4]. Pour minimiser le risque d'erreurs dans la pratique, le même examinateur a effectué tous les enregistrements durant toute la durée de l'enquête, utilisant des appareils de même marque, calibrés au préalable. Les tests ont été pratiqués entre 08 et 12 H et la meilleure des trois valeurs obtenues était enregistrée, en accord avec les recommandations d'usage. Les données ont été regroupées selon les tranches d'âge de 10 ans de 20-29 ans, 30-39 ans, 40-49 ans, 50-59 ans et 60-70 ans; pour permettre des comparaisons avec celles recueillies de la littérature dans ce domaine [4]. En fonction de l'IMC, trois catégories ont été constituées (maigre, normale, et surpoids) selon les recommandations de l'OMS pour les sujets de plus de 18 ans [38]. Deux groupes ont été considérés quant au tour de taille, avec et sans risque morbide, de même que pour le rapport tour de taille sur tour de hanche [39]. Les paramètres anthropométriques avaient été retenus comme variables indépendantes, ceux de la fonction respiratoire, comme variables dépendantes.

**Analyse statistique:** après encodage et validation, les données ont été saisies sur un ordinateur de marque *DELL* en utilisant les logiciels Epi-data 3.0 puis exportées sur une feuille Microsoft Excel 2010 et sur une page Excel logiciel GLI- 2012. Le nettoyage systématique du fichier a été réalisé au moyen du test d'exhaustivité et du test de cohérence en vue d'harmonisation et de validation des données. Les analyses ont été réalisées avec le logiciel SPSS version 23.0. Chaque valeur était présentée sous forme de moyenne ± écart-type pour les variables quantitatives et continues à distribution systématique ou sous forme de médiane pour les variables à distribution asymétrique (non-gaussienne). Les variables qualitatives avaient été décrites sous forme de fréquences relative (%) et/ou absolue (n). Pour les analyses inférentielles, la comparaison des moyennes de deux groupes a été réalisée à l'aide du test t de Student et l'analyse de variance (ANOVA) pour plus de deux moyennes. Le test exact de Fischer a été appliqué selon le cas pour comparer les proportions. Les tests de régression linéaire simple et multiple avaient été appliqués pour vérifier la corrélation entre les composantes de paramètres spirométriques dépendants (VEMS, CVF, DEP) et les variables quantitatives indépendantes (taille, poids, IMC, TT/TH, âge, tour de taille et tour de hanche). Les Chi-carré de Pearson et le test exact de Fischer selon le cas, avaient été appliqués pour comparer les proportions et vérifier la normalité de la répartition des données. Pour établir la différence de deux catégories nous avons recouru au test U de Mann-Whitney et le test de Kruskal Wallis avait été utilisé pour établir la différence de plus de deux catégories. Les limites inférieures et supérieures de la normale sont considérées: anormales lorsque les valeurs se situent en dessous du 2,5^ème^ (3^ème^) percentile et au-dessus de 97^ème^ percentile. Les valeurs anormales dont les z-scores étaient supérieurs à +4 et ou inférieurs à -4 avaient été écartés de données à l'aide de la page GLI-2012 qui nous avait fourni les z-scores des paramètres spirométriques de chaque sujet. Les valeurs normales sont comprises entre 2,5^ème^ et 97,5^ème^ percentile. Les valeurs référentielles sont proposées selon les différentes tranches des percentiles, selon l'âge et selon le sexe.

## Résultats

**Effectifs**: les effectifs de l'étude sont répartis selon le sexe et en groupes d'âge comme suit: 7443 enquêtés dont 4235 hommes et 3208 femmes; dans la tranche de 20-29 ans: 1392 (32,9%) hommes, 942 (29,4%) femmes; de 30 -39 ans: 91 8 (21,7%) hommes et 700 (21,8%) femmes; de 40-49 ans: 748 (17,7) hommes et 669 (20,9%) femmes; de 50-59 ans: 654 (15,4%) hommes, 571 (17,8%) femmes; 523 (12,3%) hommes et 326 (10,2%) femmes.

**Composition corporelle**: la répartition des sujets selon l'IMC (Tableau 1) fait apparaitre que 525 (7,0%) des sujets étaient maigres, 4329 (57,7%) avaient une corpulence normale et 2616 (34,9%) présentaient un surpoids.

**Données anthropométriques**: les caractéristiques anthropométriques et spirométriques des sujets sont consignées dans le Tableau 2. Le Tableau 2 montre la différence entre les hommes et les femmes s'agissant de la taille (T), du poids (P), tour de taille (TT) et tour de hanche(TH) ainsi que le rapport abdomen hanche (RAH). La taille médiane des hommes est supérieure à celle des femmes (171 vs 162 cm); il en est de même pour le périmètre thoracique (85 vs 82 cm.). Le poids, l'IMC, la TT, le rapport tour de taille/tour de hanche (TT/TH) et le périmètre thoracique (PT) augmentaient significativement (p<0,001) de 20 à 70 ans dans les deux sexes. Cette croissance était plus marquée entre 20 et 49 ans pour le TH et le rapport TT/TH. En revanche, on notait une diminution significative (p<0,001) de la taille de 20 à 70 ans.

**Tableau 2:** Les mesures de tendance centrale et extrême de paramètres anthropométriques et spirométriques selon les sexes

Variable	Sexe					
	Masculin			Féminin		
	Médiane	Min	Max	Médiane	Min	Max
Age (an)	37	20	70	39	20	70
Taille (cm)	171	141	196	162	141	190
Tour de taille (cm)	78	58	122	85	41	120
Tour de hanche (cm)	92	44	121	101	36	128
Poids (kg)	65	40	102	65	47	95
Rapport tour de taille/tour de hanche	0,86	0,59	1,89	0,84	0,37	2,58
Périmètre thoracique (cm)	85	44	117	82	31	121
IMC (kg/m^2^)	21,95	15,43	33,36	25,04	14,34	36,1
DEP (L/s)	6,82	4,70	14,00	5,70	4,00	12,14
VEMS (L)	3,00	1,52	5,61	2,21	1,45	4,35
CVF (L)	3,19	1,54	8,00	2,38	1,47	4,69
VEMS/CVF (%)	83	76	89	83	78	90

**Min**: minimum, **max:** maximum, **L**: litre; **L/s:** litre/seconde

**Données spirométriques**: le Tableau 3 contient les médianes des valeurs spirométriques des sujets en fonction du sexe et par groupes d'âge. Toutes les valeurs spirométriques (VEMS, CVF, DEP) diminuaient significativement avec l'âge, dans les deux sexes. Les valeurs les plus élevées ont été observées dans la tranche d'âge de 20-29 ans, également dans les deux sexes. Le Tableau 3 montre également des valeurs spirométriques médianes supérieures chez les hommes de tous les groupes, comparés à celles des femmes dans les groupes équivalents d'âge. Les écarts globaux entre les deux sexes étaient de +24,1% pour le VEMS, +23,2% pour la CVF et +29% pour le DEP. Les données spirométriques en fonction de l'IMC sont reprises dans les observations ci-dessous et elles sont différentes selon que le sujet est maigre, normal ou en surpoids.

**Tableau 3:** Valeurs spirométriques médianes par tranche d’âge et par sexe

Variables	20-29 ans	30-39 ans	40-49 ans	50-59 ans	60-70 ans	P
**Femmes**	n=942	n=700	n=669	n=571	n=326	
VEMS (L)	2,44	2,36	2,16	1,99	1,89	0,000
CVF (L)	2,59	2,52	2,35	2,11	1,98	0,000
DEP (L/s)	5,11	4,88	4,75	4,42	4,12	0,000
VEMS/CVF (%)	88	84	82	81	80	0,000
**Hommes**	n=1392	n=918	n=748	n=654	n=523	
VEMS (L)	3,39	3,09	2,94	2,66	2,24	0,000
CVF (L)	3,54	3,29	3,14	2,85	2,50	0,000
DEP (L/s)	7,63	6,97	6,56	6,25	5,81	0,000
VEMS/CVF (%)	85	83	81	80	78	0,000

**VEMS:** volume expiratoire maximum à la première seconde; **CVF:** capacité vitale forcée; **DEP:** débit expiratoire de pointe; **VEMS/CVF:** rapport de Tiffeneau

**Les valeurs médianes spirométriques** des enquêtés en fonction du sexe et de l'IMC (maigre: <18 Kgm^2^; normal: 18.0-24.9 Kgm^2^; surpoids: 25.0-29.9 Kgm^2^) se présente comme suit: VEMS(l), homme, maigre 2,86l; normal 3,06l; surpoids 2,88l; CVF(l) hommes, maigre 3,03l; normal 3,24l; surpoids 3,12l; DEF (l/s) homme maigre 6,49; normal 6,85; surpoids 6,75. VS VEMS(l), femmes, maigre 2,23; normal 2,25; surpoids 2,18; CVF(l) femmes, maigre 2,33; Normal 2,41; surpoids 2,35; DEF (l/s) femme maigre 4,79; normale 4,75; surpoids (Tableau 4).

**Tableau 4:** Valeurs spirométriques selon les percentiles (Pc), l’âge et le sexe

Variables			Les Percentiles						
Age	Sexe	Spirométrique							
			P_2, 5_	P_3_	P_4_	P_5_	P_25_	P_50_	P_97, 5_
20-29	Masculin	VEMS (l)	2,33	2,36	2,43	2,49	3,00	3,39	4,54
		CVF (l)	2,44	2,48	2,55	2,60	3,12	3,54	4,89
		DEP (l/s)	4,34	4,41	4,51	4,63	6,26	7,63	12,12
		VEMS/CVF (%)	84	84	84	84	85	85	87
	Féminin	VEMS (l)	1,93	1,94	1,98	1,99	2,19	2,44	3,31
		CVF (l)	1,96	1,99	2,00	2,02	2,31	2,59	3,56
		DEP (%)	3,62	3,66	3,76	3,83	4,52	5,11	8,58
		VEMS/CVF (%)	86	86	86	86	87	88	89
30-39	Masculin	VEMS (l)	2,11	2,14	2,22	2,29	2,74	3,09	4,42
		CVF (l)	2,29	2,33	2,38	2,42	2,90	3,29	4,74
		DEP (l/s)	4,07	4,12	4,25	4,40	5,82	6,97	11,46
		VEMS/CVF (%)	82	82	82	82	83	83	85
	Féminin	VEMS (l)	1,82	1,84	1,87	1,90	2,07	2,36	3,22
		CVF (l)	1,89	1,90	1,94	1,97	2,22	2,52	3,59
		DEP (l/s)	3,33	3,46	3,50	3,59	4,28	4,88	7,93
		VEMS/CVF (%)	83	83	83	83	84	84	86
40-49	Masculin	VEMS (l)	1,97	1,98	2,00	2,04	2,74	2,60	3,98
		CVF (l)	2,02	2,05	2,12	2,17	2,90	2,77	4,46
		DEP (l/s)	3,94	4,03	4,13	4,23	5,82	5,39	11,45
		VEMS/CVF (%)	80	80	80	80	83	81	83
	Féminin	VEMS (l)	1,76	1,77	1,80	1,82	1,98	2,16	2,93
		CVF (l)	1,80	1,82	1,85	1,87	2,07	2,35	3,34
		DEP (l/s)	3,26	3,32	3,4	3,47	4,11	4,75	7,93
		VEMS/CVF (%)	81	81	81	81	82	82	83
50-59	Masculin	VEMS (l)	1,87	1,89	1,92	1,94	2,31	2,66	3,75
		CVF (l)	1,96	1,98	1,99	2,00	2,50	2,85	4,27
		DEP (l/s)	3,55	3,71	3,92	4,06	4,96	6,25	10,73
		VEMS/CVF (%)	78	78	79	79	79	80	81
	Féminin	VEMS (l)	1,67	1,7	1,72	1,73	1,87	1,99	2,97
		CVF (l)	1,73	1,74	1,77	1,78	1,96	2,11	3,14
		DEP (l/s)	3,11	3,19	3,22	3,30	3,86	4,42	7,34
		VEMS/CVF (%)	80	80	80	80	80	81	82
60-70	Masculin	VEMS (l)	1,76	1,78	1,79	1,80	1,99	2,24	3,39
		CVF (l)	1,79	1,83	1,87	1,87	2,10	2,50	3,78
		DEP (l/s)	3,17	3,35	3,49	3,55	4,73	5,81	9,70
		VEMS/CVF (%)	77	77	77	77	78	78	79
	Féminin	VEMS (l)	1,60	1,63	1,67	1,69	1,81	1,89	2,53
		CVF (l)	1,66	1,67	1,72	1,72	1,87	1,98	2,74
		DEP (l/s)	2,99	3,00	3,01	3,04	3,65	4,12	6,76
		VEMS/CVF (%)	79	79	79	79	79	80	80

**Les valeurs spirométriques médianes**: ont été comparées entre hommes et femmes, en fonction de percentiles (P2,5 et P97,5) et de tranches d'âge. Le Tableau 4 résume les résultats observés selon les percentiles. Le Tableau 4 montre que les hommes du groupe d'âge de 20-29 ans ont un VEMS compris entre 2,33 à 4,54 Litres vs 1,93 à 3,31 litres pour les femmes; une CVF de 2,44 à 4,89 vs 1,96 à 3,56 litres, un DEP de 4,34 à 12,12 vs 3,62 à 8,58 litres/sec); le VEMS/CVF étant de 84 à 87 vs 86 à 89 %. Ce tableau indique à titre d'exemple que pour P5, 5% d'individus ont un VEMS inférieur à 2,49 litres contre 95 %, qui ont des valeurs supérieures. Dans le groupe de 30-39 ans, les valeurs sont respectivement: 2,11-4,42 vs 1,82- 3,22L (VEMS); 2,29-3,74 vs 1,89 - 3,59L (CVF); 4,07-11,46 vs 3,33 à 7,93L/s (DEP); et le VEMS/CVF identique à 83- 86 %. Le groupe de 40- 49 ans; 1,97 à 3,98L vs 1,76 à 2,93L (VEMS); CVF: 2,02 à 4,46L vs 1,80 à 3,34L (CVF); DEP: 3,94 à 11,45 vs 3,26 à 7,93L/s; le VEMS/CVF: 80 à 83 vs 81 à 83 %. Le groupe de 50 à 59 ans, respectivement: 1,87 à 3,75 vs 1,67 à 2,97 L (VEMS); CVF: 1,96 à 4,27 vs: 1,73 à 3,14L; DEP: 3,55 à 10,73 vs 3,11 à 7,34L/s (DEP); VEMS/CVF: 78 à 81 vs 80 à 82 %. Le groupe de 60 à 70 ans, respectivement 1,76 à 3,39 vs 1,60 à 2,53L (VEMS); 1,79 à 3,78 vs 1,66 à 2,74L (CVF); 2,99 à 6,76 vs 2,99 à 7,34L/s (DEP); et le VEMS/CVF: 0,79 à 0,80 vs 0,80 à 0,82%. Il ressort que la pratique d'une activité sportive impacte positivement sur les valeurs spirométriques indépendamment du sexe. Les différences sont révélées entre les valeurs spirométriques des pratiquants des sports vs non-pratiquants d'activité sportive, c'est ainsi que chez les hommes le VEMS (l) de pratiquant vs non-pratiquant est 3,36 vs 2,88 l; la CVF(L) 3,55 vs 3,07 l; le DEP (l/s) 7,92 vs 6,51 (l/s). Respectivement chez les femmes le VEMS (l) 3,00 vs 2,20 l; CVF (l) 3,12 vs 2,36 (l); DEP (l/s) 6,79 vs 4,75 l/s.

**Figure 1:**
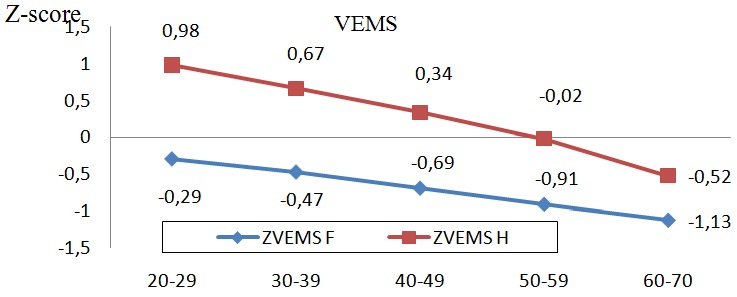
Z-score de VEMS selon la tranche d´âge entre hommes et femmes

**Figure 2:**
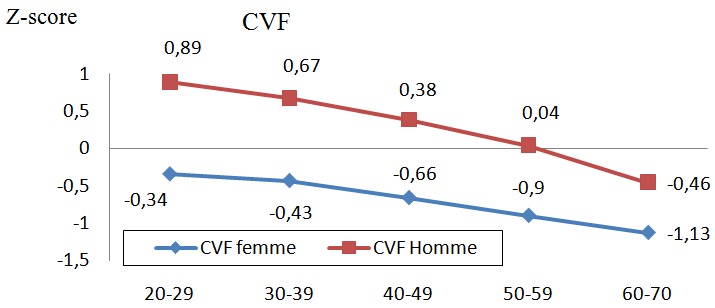
Z-score de CVF selon la tranche d´âge entre hommes et femmes

**Figure 3:**
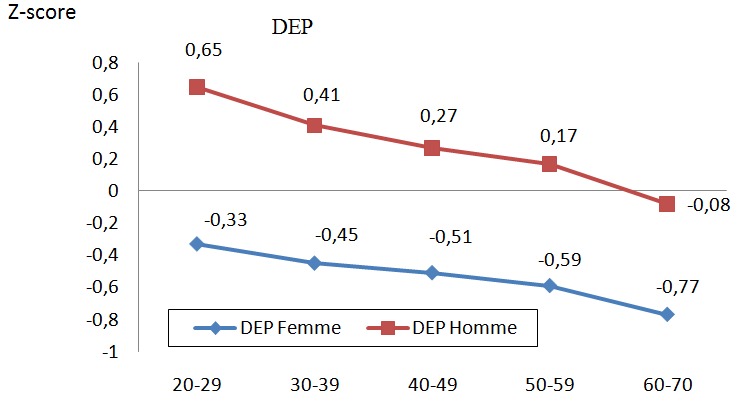
Z-score de DEP des sujets selon les tranches d´âge entre hommes et femmes

Les Figure 1, Figure 2, et Figure 3 illustrent les z-scores du VEMS, de la CVF et du DEP qui décroissent significativement d'une tranche d'âge à l'autre. Les valeurs chez les hommes sont également supérieures à celles des femmes et ce, avec un écart plus accentué en comparant la tranche de 20-29 ans à celle de 60-70 ans.

## Discussion

La présente étude visait à établir des références spirométriques dans la population saine de Kinshasa. Au total, 4235 hommes et 3208 femmes ont participé à l'étude. Les principales observations avaient révélé que les hommes avaient des valeurs du VEMS, de la CVF et du DEP supérieures à celles des femmes. Ces valeurs diminuaient avec l'âge dans les deux sexes (p<0,05). Les z-scores du VEMS et de la CVF étaient compris entre 1,5 et -1,5; celui du DEP était entre 0,8 et -0,8. Les z-scores de VEMS, de la CVF et de DEP diminuaient avec l'âge chez les sujets des deux sexes. Par ailleurs, l'âge était prédictif des différents paramètres spirométriques. L'interprétation de ces résultats doit toutefois tenir compte de deux limites principales. La première tient au type même d'étude et à sa nature transversale qui ne permet qu'un cliché temporel. La réalisation d'une enquête longitudinale et dynamique aurait été mieux adaptée dans la recherche des valeurs de références. La seconde est liée à la source des informations sur les antécédents morbides et le style de vie des sujets; ces informations n'avaient reposé que sur les déclarations des participants, sans vérification rigoureuse. Toutefois, ces limites n'affectent pas totalement la puissance des observations. Parmi les forces de la présente étude, il importe de noter le respect des recommandations quant à la réalisation des tests spirométriques. La meilleure des trois valeurs obtenues était celle enregistrée. La principale force concerne la taille de l'échantillon (n=7433Nombre), à notre connaissance, jamais atteinte dans les travaux antérieurs locaux ou d'ailleurs. En effet, l'étude de Quanjer *et al.* [40] qui s'est focalisée sur le nombre requis des sujets pour valider les valeurs respiratoires de référence rapporte une population d'au moins 150 hommes et 150 femmes, pour éviter les biais dus à une erreur d'échantillonnage. Rajaee M *et al.* [31] au Ghana rapportent une taille de 172 sujets, Shamssain *et al.* [41] en Lybie 208 sujets, Omar A *et al.* [42] en Omani 561 sujets, Pefura Y *et al.* [43] au Cameroun 1357 sujets, Mohamed O *et al.* [44] en Arabie Saoudite 621 sujets, Ratomaharo J *et al.* [29] au Madagascar 2491 sujets et Musafiri *et al.* [32] au Ruanda 1824 sujets. La troisième force de l'étude est l'établissement des équations de prédiction des valeurs spirométriques en utilisant la méthode statistique de régression multi variée PLS. Le choix de ce modèle mathématique s'appuie sur quatre critères: la variation étroite pour expliquer la variable dépendante, la détermination du coefficient R2, la constance de la déviation standard résiduelle (DSR).

Notre étude a établi des valeurs spirométriques de référence mesurées sur un échantillon fortement représentatif d'une population congolaise bantoue d'adultes sains. Nos résultats confirment que la CVF, le VEMS et le DEP pour un sujet dépendent de l'âge, du sexe, du niveau de pratique sportive et de l'ethnie. Par rapport à l'âge cette différence est due aux transformations de la morphologie de 20 à 70 ans. Celles-ci sont caractérisées par une augmentation des volumes pulmonaires de 20 à 50 ans puis une baisse progressive au-delà de 50 ans, en suivant la courbe d'évolution de la taille et du poids. L'augmentation significative de la CVF et du VEMS peut s'expliquer par le développement du tissu pulmonaire, en rapport avec le développement musculo squelettique de la cage thoracique et l'accroissement des espaces intercostaux. Cette observation validée dans les deux sexes a été décrite dans d'autres études africaines [26-36]. Par exemple, l'étude de Tabka Z *et al.* en Tunisie [27] montre que les facteurs morphologiques (sexe, taille, poids, développement de la poitrine) sont plus fortement associés aux variables ventilatoires pour les deux sexes. Ces observations rejoignent aussi celles de Dufetel et al chez les adultes sénégalais [28].

Les différences interraciales des valeurs spirométriques ont été décrites antérieurement entre caucasiens et non caucasiens [20-24]. À travers le monde, les valeurs spirométriques étaient différentes entre caucasiens et non-caucasiens, notamment: Hankinson (USA) 1996, dans la tranche d'âge de 20-25 ans 4,44l (VEMS), 5,26l (CVF); Helena (SWEDE) 2012, âge 20-39 ans 4,18 ± 0,78 5,30l (CVF) , 78,8 ± 0,67 % (VEMS / CVF); Mohamed G (ARABIE S) 2012, 3,64 ± 0,448 (VEMS), 4,50l CVF, 80,9 ± 3,65 VEMS / CVF (%); Bashir (SOUDAN) 2010, 2,96l (VEMS), 3,26l (CVF); Kamanga MB (RDC) 2011, âge, 18-25 ans 2,55 ± 0,44L (VEMS), 2,74 ± 0,51L (CVF); notre étude (2018), tranche d'âge: 20-29 ans 3,39L (VEMS), 3,54L (CVF), 85% (VEMS/CVF) [40,41]. Les faibles valeurs spirométriques relevées chez nos sujets par rapport à celles notées chez des adultes dans les études européennes ([1-9] [19-24]) et américaines [9-16] pourraient s'expliquer tout au moins partiellement par les conditions environnementales et nutritionnelles de précarité en Afrique Subsaharienne. L'UNICEF rapporte des statistiques alarmantes quant à la morbimortalité liée aux conditions d'hygiène insuffisante, renforcées par les guerres et les migrations déstabilisatrices des populations (rapport OMS, 2010). Notre étude corrobore les résultats de plusieurs travaux antérieurs qui ont démontré des différences spirométriques entre groupes ethniques (caucasiens et non caucasiens) et entre hommes et femmes. Cette disparité est ainsi constatée dans tous les continents et entre les continents. Les études publiées depuis 1970 jusqu'à ces jours ont montré l'influence des facteurs anthropométriques sur les valeurs spirométriques, notamment la supériorité des valeurs caucasiennes sur celles des populations non caucasiennes. C'est le cas des enquêtes de Montero *et al.* (1970), Dedoyard *et al.* [34], et Ghesquiere *et al.* en 2009 [35] qui ont rapporté ces différences interraciales en milieu congolais. Des études menées ailleurs en Afrique [25-34] soutiennent cette différence. Dans le présent travail, le sexe apparaît comme un autre facteur discriminant; en effet, les valeurs spirométriques médianes des femmes sont inférieures à celles des hommes (valeurs VEMS 2,44L pour femmes vs 3,39L pour hommes (Tableau 3). La composition corporelle différente entre les deux sexes pourrait influer sur la mécanique thoracique et l'expansion thoracique limitée par la plus grande masse grasse chez la femme. La participation des muscles thoraciques et du diaphragme sur la capacité ventilatoire est un fait bien connu [3].

L'étude établi une différence entre sujets pratiquants et non pratiquants d'une activité sportive. Nos observations rejoignent celles d'autres auteurs concernant la supériorité des valeurs spirométriques des sportifs, plus élevées que celles observées dans cette étude. Durmic T *et al.* (2015) au Brésil avait trouvé 17% différence entre sportif et non-sportif [45] tandis que la différence dans notre étude était de 14,7%. Par ailleurs, Quanjer *et al.* (1979) en Hollande avait trouvé la CVF des sportifs européens à 6,25 L, Katch *et al.* (Américain) 1970, la CVF sportive était de 6,00 L; Helena Bck *et al.* (Suède) 2015, la CVF moyenne des sportifs était 5,76±0 ,76 l; alors que Ghesquiere J *et al.* (2009) en RD Congo avait trouvait la CVF des joueurs de football à 4,40 L (35) tandis que notre étude à Kinshasa RDC (2018) avait trouvé la CVF des sportifs à 3,55L. Ce fait est intelligible, surtout pour le DEP qui est directement « effort dépendant » mais également pour le VEMS et la CVF où l'effet du sport est retrouvé. Lazovic *et al.* [46], de même que Troosters *et al.* [47], ont rapporté un constat similaire. Ce dernier groupe a montré que la distance de marche chez l'individu sain était liée à l'âge, au sexe et à la taille, trois facteurs qui interviennent dans la détermination des volumes ventilatoires. Quoique non abordé dans ce travail, il est un fait connu que la pratique de l'activité sportive induit une augmentation de la consommation maximale d'O_2_, et influe donc sur la performance ventilatoire; on peut dès lors mieux envisager l'impact de la pratique sportive sur le travail ventilatoire chez le sujet pratiquant une activité sportive. La réponse ventilatoire à l'exercice suppose l'augmentation de la fréquence ventilatoire et aussi du volume d'air mobilisé durant chaque mouvement respiratoire [47]. Cela implique une intervention plus active de la musculature inspiratoire (muscles intercostaux, muscles scalènes et sterno-cléido mastoïdiens). Ces facteurs, en plus de la taille et de la masse musculaire [5], pourraient aussi expliquer les différences retrouvées entre hommes et femmes pratiquant le sport. S'agissant de la corrélation des volumes ventilatoires avec l'âge, nos observations corroborent celles d'autres études [8-34]; c'est le cas d'un travail mené au Maroc par Maouni, qui a ressorti l'influence de l'âge sur le DEP chez l'adulte [33]. Une explication possible de ce constat, c'est la forte dépendance des débits respiratoires du morphotype de l'individu et du type de sport pratiqué.

## Conclusion

La présente enquête, tout en montrant des différences liées au genre, à l'âge, aux données anthropométriques, et à la pratique ou non d'une activité sportive a permis d'établir des valeurs spirométriques de base en fonction des percentiles, dont la validation par des enquêtes multicentriques et dans tout le pays pourrait permettre l'élaboration d'un référentiel de données spirométriques chez l'adulte sain en R D Congo.

### État des connaissances actuelles sur le sujet

- L'influence des paramètres anthropométriques sur les valeurs spirométriques est connue mais à partir des populations blanches. Peu ou pas de données en Afrique subsaharienne existent;
- l'influence de l'âge, du sexe sur les valeurs spirométriques est connue;
- la différence des valeurs spirométriques entre hommes et femmes est connue.

### Contribution de notre étude à la connaissance

- Elle permet d'élaborer un référentiel des valeurs spirométriques en fonction de l'âge, du sexe, et de la pratique ou non d'une activité physique. Les analyses sont faites selon les percentiles comme recommandé par les sociétés savantes;
- Cette étude est la première dans le pays et elle inclut plus de 7000 sujets;
- Elle apporte aux professionnels de la santé et à d'autres groupes de la population locale un instrument de mesure et de comparaison.

## Conflits d’intérêts

Les auteurs ne déclarent aucun conflit d’intérêts.
